# Supplementary material for: Curvature-induced expulsion of actomyosin bundles during cytokinetic ring contraction
Source: eLife. 2016 Oct 13;5:e21383. doi: 10.7554/eLife.21383 (PMC5077295; doi:10.7554/eLife.21383)
Supplement: Supplementary file 1. — DOI: http://dx.doi.org/10.7554/eLife.21383.015 [file elife-21383-supp1.docx]

**Table S1**. Fission yeast strains used in this study

| Strain | Genotype | Source |
| --- | --- | --- |
| MBY10121 | *S. japonicus ade6sj*-domE *ura4sj*-D3 h+ | [Gu et al., 2015] |
| MBY10152 | *S. japonicus cdc25*-D9::*ura4+*::*kanMX6* *rlc1-GFP*::*kanMX6* h+ | [Gu et al., 2015] |
| MBY10194 | *S. japonicus cdc15-GFP*::*ura4+*::*kanMX6* *ade6sj*-domE *ura4sj*-D3 h+ | [Gu et al., 2015] |
| MBY10339 | *S. japonicus GFP-rng2*::*ura4+* | [Gu et al., 2015] |
| MBY10355 | *S. japonicus LifeAct-GFP*::*ura4*+ | [Gu et al., 2015] |
| MBY192 | *S. pombe ura4-D18 leu1-32 h-* | Lab collection |
| MBY5763 | *S. pombe cdc25-22 Rlc1-3GFP::kanMX6 h+* | Lab collection |
| MBY6656 | *S. pombe Pact1-LifeAct-GFP::leu1+ ura4-D18 leu1-32 h-* | Lab collection |
